# Microfluidic In-Flow Decantation Technique Using Stepped Pillar Arrays and Hydraulic Resistance Tuners

**DOI:** 10.3390/mi10070471

**Published:** 2019-07-15

**Authors:** Gangadhar Eluru, Pavan Nagendra, Sai Siva Gorthi

**Affiliations:** Optics and Microfluidics Instrumentation Lab, Department of Instrumentation and Applied Physics, Indian Institute of Science, Bangalore 560012, India

**Keywords:** in-flow decantation, self-sheath generation, microfluidics, sheath-free flow focusing, plasma separation, particle enrichment

## Abstract

Separating the particles from the liquid component of sample solutions is important for several microfluidic-based sample preparations and/or sample handling techniques, such as plasma separation from whole blood, sheath-free flow focusing, particle enrichment etc. This paper presents a microfluidic in-flow decantation technique that provides the separation of particles from particle-free fluid while in-flow. The design involves the expansion of sample fluid channel in lateral and depth directions, thereby producing a particle-free layer towards the walls of the channel, followed by gradual extraction of this particle-free fluid through a series of tiny openings located towards one-end of the depth-direction. The latter part of this design is quite crucial in the functionality of this decantation technique and is based on the principle called wee-extraction. The design, theory, and simulations were presented to explain the principle-of-operation. To demonstrate the proof-of-principle, the experimental characterization was performed on beads, platelets, and blood samples at various hematocrits (2.5%–45%). The experiments revealed clog-free separation of particle-free fluid for at least an hour of operation of the device and demonstrated purities close to 100% and yields as high as 14%. The avenues to improve the yield are discussed along with several potential applications.

## 1. Introduction

Microfluidics, in its immense potential to offer miniaturization, cost-effectiveness, precision, automation, and the use of ultra-small quantities of samples, is rapidly expanding into areas of health-care, water treatment, soil testing, biomedical research, chemical and biological sciences. In several applications involved in these areas, separation of suspended particles from the sample fluid is a must as a pre-preparatory step for further investigation/research. As most of the research in microfluidics is oriented towards health-care, the foregoing discussion presents the importance and latest developments of separation process in this area. In health-care, the majority of diagnostic tests are performed on body fluids such as blood, as the biomarkers associated with most of the body’s medical conditions can be found in them.

Blood is an important body fluid, that is responsible for the delivery of nutrients, oxygen to the cells and takes away metabolic waste from the cells. Its main constituents are plasma (≈55% by volume) and blood cells (≈45% by volume). Approximately 92% by volume of plasma is water and contains dissipated proteins, glucose, mineral ions, carbon dioxide etc. The blood cells are majorly red blood cells (RBCs), white blood cells, and platelets. Many markers associated with changes in health reflect as either a change in the composition of plasma or blood cells. Blood-based tests can be broadly categorized into plasma/serum-based tests and blood cell-based tests.

The majority of plasma/serum-based tests require the separation/removal of blood cells from the whole blood as the cellular debris interferes with most of plasma/serum-based tests. The standard laboratory technique of cell separation from whole blood is based on centrifugation, which is very cumbersome, time consuming (about 30 min) and requires skilled personnel to operate. Microfluidics based devices for plasma separation can be majorly classified into active and passive. The devices based on active techniques use some form of forces such as dielectrophoretic, electrohydrodynamic, electro-osmotic, centrifugal, and acoustic to achieve the desired cellular separation [1,2,3,4,5,6,7]. The major disadvantages associated with these techniques are the inability to provide high quality plasma at higher hematocrits, complexity in fabrication and integration, and the typical dependence on bulky external power supplies.

Passive techniques take leverage on one or a combination of the parameters, such as the use of filters, device geometry, inertial or Dean’s effects, gravity, and biophysical effects which include the Fahraeus effect and the Zweifach-Fung bifurcation law to achieve the desired separation. The techniques of plasma separation based on filtration suffer with major problems of clogging due to the very small pore size of filters [8]. Plasma separation designs based on geometrical and biophysical effects [9,10,11,12,13] do not cause clogging problems in general, however, they provide high purity plasma only at lower hematocrits and/or smaller flow rates, thereby limiting their use for rapid diagnosis of majority of biochemical tests [14]. Inertial and Dean’s effects were utilized to separate plasma by pumping the blood at higher flow rates and were demonstrated to produce high purity plasma only at lower hematocrits [15]. Several other passive variant techniques that demonstrated plasma separation have been extensively reviewed by Han et al. and Siddhartha et al. [16,17]. However, the production of high purity plasma at larger hematocrits (from whole blood) at optimum flow rates (without causing hemolysis) in higher yields, in a simple to fabricated device, is still a challenge.

Most of the blood-cell based tests using microfluidics to analyze blood cells in flow on the flow cytometric principles of imaging or scattering. One of the crucial requirements of these microfluidic devices is flow focusing of the cells into a plane or a line for interrogation widely known as flow focusing. Most of the flow focusing techniques employ external sheath fluid to focus these cells. The efforts to generate sheath-free flow focusing have led to the development of both passive and active techniques. Due to the conventional drawbacks associated with active techniques, such as complexity in fabrication and integration, bio-cellular incompatibility and the requirement of bulky and expensive power supplies, passive techniques remained as an attractive choice for microfluidic based diagnostics.

Most of the passive techniques operate at high flow rates by utilizing either Dean’s effects or inertial effects and are not suitable for imaging based applications [18,19,20]. One of the passive techniques that operate at low flow rates uses pillars to achieve the separation of particle-free fluid from the particles and reuse them to accomplish sheath-free flow focusing [21]. The proposed pillar-based design works well for rigid particles of larger size than the spacing between the pillars, but is not suitable for separating deformable particles, such as red blood cells from the fluid. To use such a device for blood cell (particle size ranges from 2 μm to 30 μm) separation and focusing requires fabricating the pillars with a spacing smaller than 2 μm while keeping the height of the pillar (and channel) large enough to accommodate the largest particle (30 μm), which is highly difficult to achieve using regular techniques of photolithography and soft lithography. This pillar design limits its applicability to only rigid particles and could not answer the problem of clogging due to the uncontrolled fluid flow through the pillar gaps. This leaves the problem of providing a clog-free passive separation design that is simple to fabricate, that can work for a wide range of particle ranges and flow rates, as open ended and demand solutions.

This paper presents a technique that provides solutions to the aforementioned problems in a single device. The presented design can separate the particles from the fluid for a wide range of flow rates, particle sizes and concentrations in very high purity and yield, in a single polydimethylsiloxane (PDMS) layer device, through the use of the principle termed as wee-extraction. The device consists of stepped pillar arrays (different from the regular pillar arrays due to their size and placement) and hydraulic resistance tuners in a unique combination to control the fluid flow through the pillar gaps in the stepped pillar array. This unique combination offers the proposed technique needed that has advantages over the other techniques to accomplish decantation in high purity and yield. The applicability of this technique for low flow rates as well as high flow rates and for wide variety of particle sizes without clogging was demonstrated. The high purity (100%) and yield (14%) of the particle-free fluid separated, and the applicability, efficient working of the technique even at higher particle concentrations (45%) demonstrates the potential of the proposed technique and its practical utility. The principle of operation of this technique was demonstrated using beads (5 μm), platelets, and blood of various particle concentrations (hematocrits). This novel proposed technique in combination with any simple technique to fabricate a 3D flow focusing device that uses external-sheath fluid [22] can be used to develop a sheath-free flow cytometer that meets the needs of blood-cell based tests. Another important feature of this design is simplicity in fabrication, as it involves fabricating a master mold only once and the subsequent device fabrication can be done in a single step without the requirement of complicated alignment procedures, although the design involves multiple heights. The following sections present the theory and principle of operation, simulation and experimental results along with their consequent implications.

## 2. Theory, Simulations and Experiments

### 2.1. Theory and Principle of Operation

**Figure 1:**
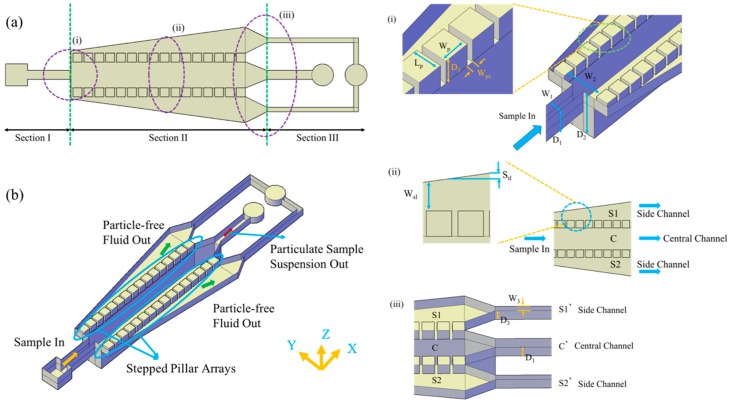
Schematics representing the proposed decantation device. (**a**) Schematic of the proposed microfluidic device. (**b**) An isometric view of the device schematic to better visualize the features. (**i**), (**ii**), and (**iii**) represent the representative schematic images at the appropriate locations.

**Figure 2:**
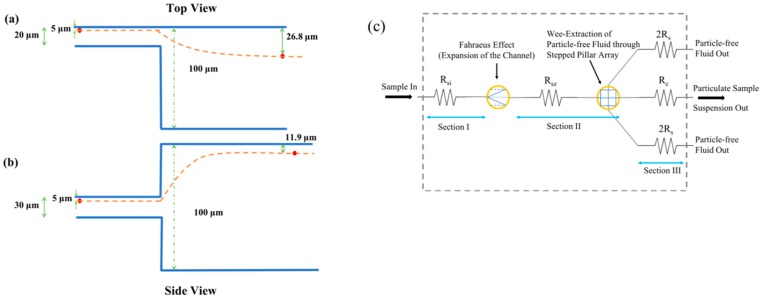
Schematics illustrating the movement of particle due to the expansion of the channel (Fahraeus effect) and a hydraulic resistance model of the device. (**a**,**b**) represent the schematics illustrating the movement of the particle due to the expansion of the channel. Motion of the particle which was originally moving at 5 μm away from the top and side walls prior to expansion of the channel is shown in (**a**,**b**), as seen in side and top views, respectively. The numbers shown in the figure were obtained from simulation results. (**c**) Schematic representing the hydraulic resistance model of the device. *R_si_*, *R_sz_*, *R_c_* and 2*R_s_* are the hydraulic resistances of the appropriate sections/channels of the device shown in Figure 1. New symbols were used to represent the phenomena happening on the particles due to Fahraeus effect and wee-extraction.

The device design and its operation can be well understood by artificially dividing the whole design into three sections namely Section I, Section II and Section III as shown in Figure 1a. Section I consists of the sample input and channel of depth *D_1_* for the sample inflow. Section II is further subdivided into central channel C and side channels S1 and S2, each having depths *D_2_* and *D_3_*, respectively. The meeting point of Section I with Section II has a transition of depth from *D_1_* to *D_2_* and width *W_1_* to *W_2_*. The side channels S1 and S2 are identical and are placed symmetrically on either side of central channel C. These side channels consist of series of pillars with spacing between them as *W_ps_*. This configuration of pillar array with height (*D_3_*) different from that of channel C (*D_2_*) and placed towards one end of the channel (in the height direction) is termed the stepped pillar array. A schematic better illustrating the stepped pillar arrays is shown Figure 1b. The central channel depth *D_2_* and width *W_2_* are chosen larger than the sample channel depth *D_1_* and width *W_1_*, to facilitate the redistribution of sample particles present inside the sample solution along the depth and width directions. For a dilute suspension of particles, as the sample passes from the sample channel to the central channel, the average separation of particles and the average distance of the particles from the top, bottom and side walls increases due to the expansion of the channel in the depth and width directions. Schematics illustrating the same are shown in Figure 2a,b. This increment in the distance of the particles from the walls leaves the room for a large quantity of particle-free fluid to be closer to the walls. This is the first essential accomplishment of the proposed design. The separation of this particle-free fluid, stepped pillar array design along with hydraulic resistance tuners is proposed.

The stepped pillar array consists of pillars of size *L_p_*, *W_p_* and *D_3_* as shown in Figure 1. The width of the pillars (*W_p_*), the spacing between them (*W_ps_*), and the number of pillars (*N*) in each side channel decide the overall size of the device. The side channels lateral spacing *W_sl_* was continuously increasing from the start of the pillars until the end in steps of *S_tl_* per period. The purpose of using the pillars with constant spacing *W_ps_* between them and a variable lateral spacing *W_sl_* is to extract a small and controllable quantity of fluid through the pillar gaps continuously. If there is a zero step increment *S_tl_*, it is expected to have no further accumulation of fluid through the side channels, due to the pressure of the already existing fluid in that region, except towards the end of Section II. Hence, to have a larger quantity of fluid to accumulate in the side channels, there is a need to have some nonzero *S_tl_*.

The spacing between the pillars *W_ps_* and the height of the pillar *D_3_* define the cross-section for the fluid flow through the pillar gaps, hence providing a measure for fluid extraction through them. The smaller the pillar gap cross-section, the better the control is over the quantity of fluid drawn through the pillar gaps, for a given Section III design. Designing this volume of extraction through each of these gaps smaller than the volume of the critical particle to be separated, ensures the particles are completely separated out of the fluid. The volume of the fluid drawn through pillar gaps (*V_pg_*) depends on the cross-sectional area of the pillar gap (*W_ps_* × *D_3_*), the average velocity of flow across the pillar gaps (*v*), and the average velocity of flow near the pillars in the direction of overall fluid flow (*u*) and is given by *V_pg_* = *W_ps_* × *D_3_* × *v* × (*W_ps_*/*u*). Smaller *V_pg_* can be obtained by making the terms *W_ps_*^2^ × *D_3_* and *v*/*u* smaller. The ratio *v*/*u* is coupled to the cross-section of pillar gaps and Section III of the design. To obtain a smaller *V_pg_*, in principle, *W_ps_* or *D_3_* or *v/u* or any combination of them can be chosen arbitrarily small. Regarding the fabrication difficulties, *W_ps_* and *D_3_* can be made small enough to conveniently fabricate and *v/u* can be manipulated by controlling the Section III design. This ensures a smaller quantity of fluid withdrawal through the pillar gaps. This process of extracting smaller quantities of fluids through the pillar gaps is defined as the principle of wee-extraction.

Section III consists of a central channel C’ and the side channels S1’ and S2’ with hydraulic resistance *R_c_* and *R_s_*, respectively. These side channels S1’ and S2’ are the hydraulic resistance tuners of the proposed device, which can be changed by changing the length or cross-sectional parameters of these side channels. *R_s_* is the hydraulic resistance of the two side channels S1′ and S2′ combined. As these two side channels are identical, the hydraulic resistance of each side channel can be 2 × *R_s_*. The main function of this Section III design is to control the ratio of the fluid that gets drawn through the side channels with respect to the central channel, which in turn is controlled by the ratio of hydraulic resistances of the central and side channels C’, S1’, and S2’. The flow rate ratio of side channels S1’ and S2’ (combined total flow rate = *Q_s_*) and that of central channel C’ (*Q_c_*) is given by *Q_s_*/*Q_c_* = *R_c_*/*R_s_*. The side channel flow rate *Q_s_* in terms of the sample flow rate *Q* is given by *Q_s_* = (*Q* × *R_c_*)/(*R_c_* + *R_s_*). The above analysis is approximate and can be used to get only a qualitative understanding of the separation process. In the above analysis, it is presumed that the Section II device parameters do not have any significant effect on the flow rates *Q_s_* and *Q_c_*, except for the distribution of flow inside Section II. This was proven to be true through both simulations and experiments as presented in subsequent sections.

The summary of the whole functionality of the device is depicted in the hydraulic resistance model of the device as shown in Figure 2c. The hydraulic resistances of various sections and channels of the devices are modelled as resistors and the effect of phenomena, such as Fahraeus effect and wee-extraction through stepped pillar array, are represented with symbols. The transition from Section I of the design to Section II of the design leading to a depth and width increment has resulted in bringing more quantity of particle-free fluid towards the walls of the channel (Fahraeus effect). Sections II and III of the design together bring out the wee-extraction of the fluid through the stepped pillar array from the suspension. This latter process of wee-extraction first involves extracting the particle-free fluid into the side channel that was made available due to the Fahraeus effect, and subsequent extraction of the particle-free fluid that is present in the suspension, but was not made available as particle-free layers due to Fahraeus effect. This extraction of particle-free fluid through the stepped pillar array happens in Section II, but being controlled by the hydraulic resistances of Section III.

For scenarios that involve lower concentration of sample particles, the former process (Fahraeus effect) creates larger quantities of particle-free fluid near the walls. A suitable choice of parameters for the latter process can facilitate the extraction of this fluid into the side channels. This use of the Fahraeus effect helps in having a much greater yield of separation of particle-free fluid, when used with dilute suspension of particles, which in turn can be used for sheath-free flow focusing of particles. However, as the particle concentration increases, the Fahraeus effect may not be able to generate large quantities of particle-free fluid near the walls. This leaves the technique to solely depend upon the latter process to separate the particle-free fluid from the suspension. This can be accomplished by choosing the wee-extraction volume across the pillar gaps to be smaller than the critical particle volume to be separated. This process of extracting the particle-free fluid (wee-extraction) facilitates a possibility of extracting the particle-free fluid in very high purity and in modest yields. This technique offers a scope for further enhancing the yield through cascading as discussed in subsequent sections.

### 2.2. Device Fabrication

**Figure 3:**
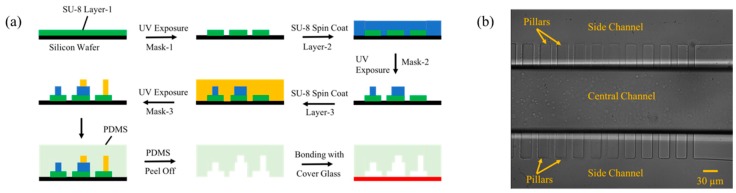
Schematics illustrating the multi-step variable height lithography and a micrograph of the fabricated device. (**a**) Schematic represents the various steps involved in achieving the 3 different heights into the Master mold and then transferring them into the PDMS device in a single shot. (**b**) Micrograph of the PDMS device that was fabricated and shows the image of the device towards the end of Section II.

The design of the device is such that the three different depths required can be obtained into a single layer of PDMS, and hence is very simple to fabricate. The three heights on the Master were prepared by the usual process of multi-step variable height optical lithography using three SU-8 photoresists 2005, 2015 and 2100 as illustrated in Figure 3a. The first layer was fabricated by spin coating SU-8 2005 photoresist and exposing it to ultraviolet (UV) light using a mask design that contains the stepped pillar arrays (Mask-1 Supplementary Information Figure S1). This layer is developed using the developer solution and contains the stepped pillar arrays and the hydraulic resistance tuners. The second layer was fabricated on top of the first layer by spin coating SU-8 2015 and exposing it to UV light using the mask design-2 (Supplementary Information Figure S2). The second layer ensures the inlet channel is of desired height to intake the particles without clogging. The third layer is further fabricated by spin coating SU-8 2100 and exposing it to UV light using the mask design-3 (Supplementary Information Figure S3). This ensures the central channel height is much larger than inlet channel to facilitate the Fahraeus effect. The resulting depths *D_1_*, *D_2_*, and *D_3_* were measured using the Dektak surface profiler and were found to be 20 μm, 74 μm, and 4.5 μm respectively. PDMS devices were further fabricated from the Master using the standard process of soft lithography. The micrograph of the fabricated device that was taken towards the end of Section II that depicts the stepped pillar arrays, central and side channels is shown in Figure 3b.

### 2.3. Sample Preparation

Fresh venous blood was collected from healthy subjects in vacutainers containing EDTA (Ethylenediaminetetraacetic acid) anticoagulant. The blood was diluted with phosphate buffer saline (PBS) (135 mMNaCl, 2.7 mM KCL, 10 mM Na_2_HPO_4_, 2 mM KH_2_PO_4_ and pH adjusted to 7.4) accordingly as per the needed hematocrit. For experiments with platelets, the undiluted blood was centrifuged at 3000 rpm for 20 min and the resulting supernatant was diluted by a factor of 2 by adding PBS. Further, 5 μm polystyrene bead solution was prepared by diluting the 10 μL of the raw suspension obtained from Sigma-Aldrich in 1ml of water.

### 2.4. Simulations

As the exact design that was used for the experiments is complex and computationally intensive, the simulations were performed on a simpler model that captured the essence and provides insights into the behavior of particle separation. This design consists of Section I of the model discussed above, but of smaller length. The Section II consists of a smaller number of pillars than the actual device ranging from 50 to 200, but similar other parameters. The parameters used for the simulations are *D_1_* = 20 μm, *D_2_* = 100 μm, *D_3_* = 5 μm, *W_ps_* = 10 μm, *W_p_* = 20 μm, *L_p_* = 40 μm. The Section III design has kept the hydraulic resistance ratios of the side channel (S’) to the central channel (C’), abbreviated as *SCRR* (side channel to central channel resistance ratio), fixed while performing the simulations with respect to variations of other parameters. The schematic of the design that was used for simulations is shown in Supplementary Information Figure S4. The fluid dynamic simulations were performed on COMSOL multi physics software version 5.2 using the inbuilt laminar flow module. The fluid for flow inside the design was chosen to be water and the mesh type was chosen to be tetrahedral and extremely fine. The wide varieties of simulations were performed by changing relevant parameters and the results have been presented in the results and discussion sections.

### 2.5. Experimental Procedure

**Figure 4:**
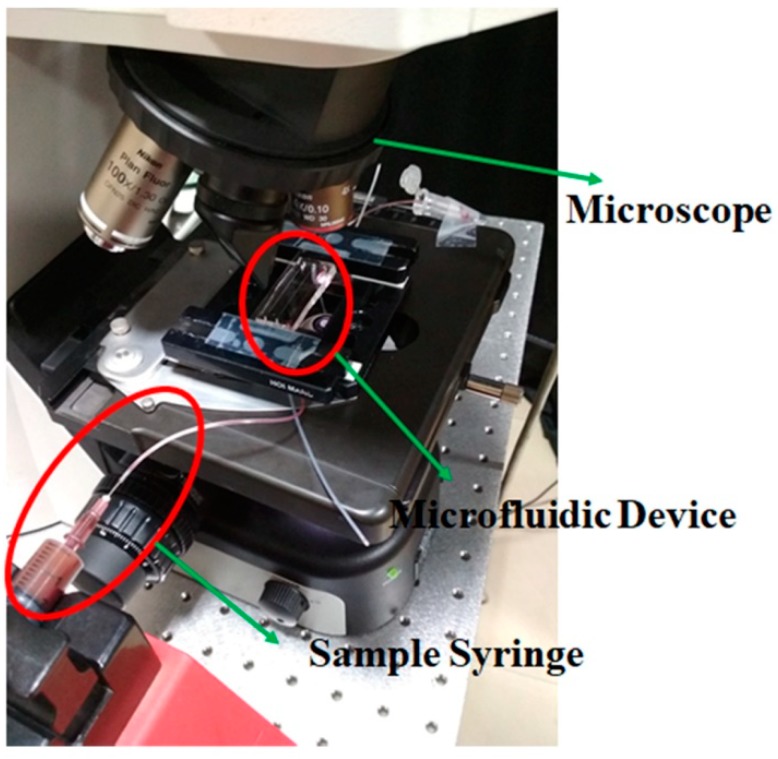
Experimental set-up that was used for the validation of proposed microfluidic technique.

Regarding the practical applicability of the proposed technique for biological applications, experimental characterization was performed on the fabricated devices by pumping blood at different hematocrit (*hct*) levels. The blood cells in flow were imaged using a Nikon microscope, 10× micro objective and Pike camera as shown in Figure 4. The number of blood cells escaping through the side channels were counted to quantify the purity of the side fluid collected. The purity of the side fluid is defined as,

$$\mathrm{Purity}\;=\;\left[\frac{\left(N_{sampl}\;-\;N_{s}\right)}{N_{sampl}}\right]\;\times \;100\%,$$

where *N_sampl_* is the total number of particles expected to enter the device per second and *N_s_* is the total number of particles entering the side channels S1 and S2 per second. *N_sampl_* is computed based on hematocrit, flow rate (*Q*) and is given by *N_sampl_* = *Q* × *hct*, whereas *N_s_* is computed based on the images of the cells entering S1 and S2. The images that were acquired have been post processed using morphological operations tool box available in MATLAB and a custom written code to obtain the number of cells entering S1 and S2 (Supplemenatry Information).

For all the experiments, the fluid from the side channels and fluid from the central channel were collected separately into vials using polyethylene tubing. The quantification of the volume of the fluid extracted in side channels was performed by measuring the length changes of the fluid inside the polyethylene tubing attached to side channels and multiplying with the inner area of the cross-section of the tubing. The measurement has a precision of less than 5% in determining the volume of fluid collected in side channels. This is because every measurement of the length change inside the polyethylene tubing for volume estimation was larger than 2 cm and the error in measuring the length change was less than 1 mm.

The above experimental procedure was carried out by varying a number of parameters to understand their effects. The parameters include the side channel to the central channel resistance ratio (*SCRR*), the hematocrit value (*hct*), the flow rate (*Q*) and time. Another study was carried out to characterize the purity of sheath collected by varying the particle size. Platelets and 5 μm beads were considered for these experiments in place of RBCs. Both these particle suspensions were then pumped into the microfluidic device at 100 μlh^−1^ and imaged for estimating the purity of the particle-free fluid collected.

## 3. Results and Discussion

### 3.1. Simulations

From the theory, it is expected that the successful development of a device should decant particles below a critical particle volume, understand the flow across all the pillar gaps and know the parameters that control the volume of the net fluid being drawn through the side channels S1′ and S2′. The simulations were performed to understand the effect of various parameters of Sections II and III on the net fluid being drawn through the side channels S1′ and S2′ and the effect of Section II parameters on flow uniformity across the pillar gaps. The results of these simulations along with the respective conclusions are presented below.

#### 3.1.1. Effect of Section II Parameters [Height of Pillars (*D_3_*), Spacing between the Pillars (*W_ps_*), Number of Pillars (*N*), Slope of the Side Channel (*S_slp_*), and Flow Rate (*Q*)] and Section III Parameters [Side Channel to Central Resistance Ratio (*SCRR*)] on Side Channel to Central Channel Flow Rate Ratio (SCF)

**Figure 5:**
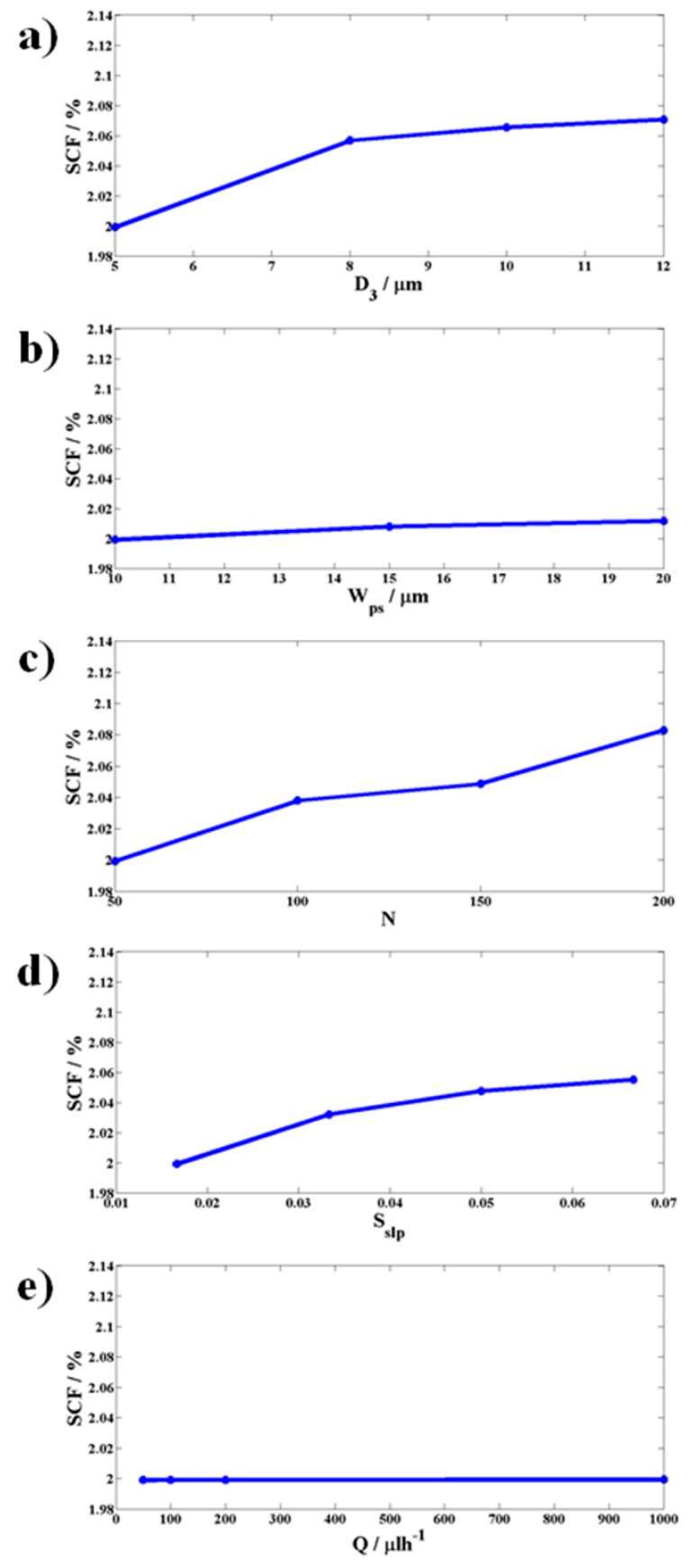
Each panel reports the trend of *SCF*. The simulation results showing the effect of parameters of Section II, namely (**a**) height of the pillar (*D_3_*), (**b**) spacing between the pillars (*W_ps_*), (**c**) number of pillars (*N*), (**d**) slope of the side channel S (*S_slp_*), and (**e**) the sample flow rate (*Q*) on the side to central channel flow rate ratio (*SCF*), respectively. The results indicate there is no significant effect of the parameters of Section II and the flow rate on *SCF*.

**Figure 6:**
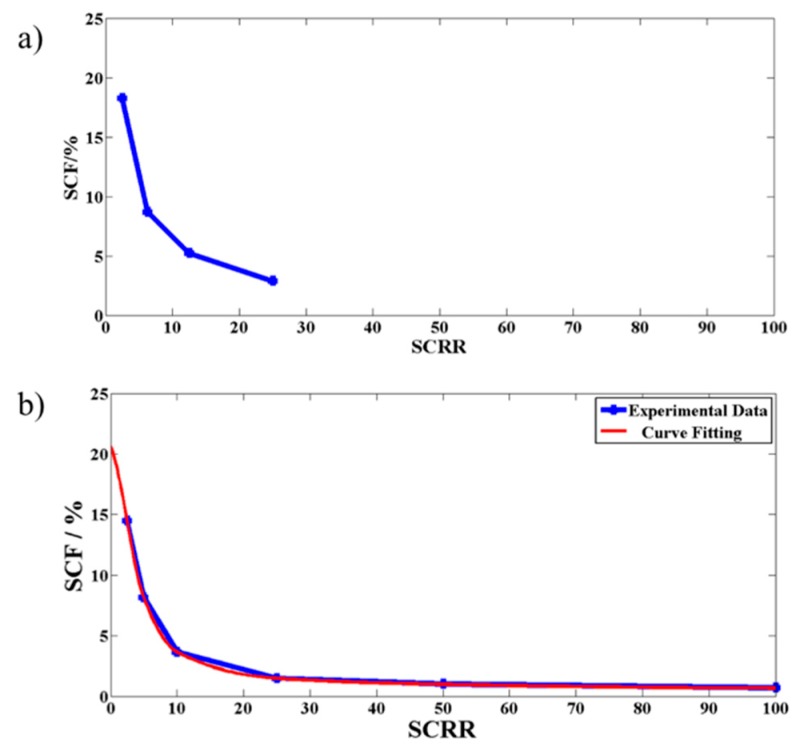
The effect of the side to the central channel hydraulic resistance ratio (*SCRR*) on the side to the central channel flow rate ratio (*SCF*). (**a**) The simulation results demonstrating the effect of *SCRR* on *SCF*. (**b**) The experimental results showing the variation of *SCF* with respect to *SCRR*.

The simulations were performed on a device model that was presented in a simulations section with parameters *D_1_* = 20 μm, *D_2_* = 80 μm, *N* = 50, and with all other parameters as specified in Figure 5 and Figure 6a accordingly.

The side channel slope *S_slp_*, the side channel to the central channel hydraulic resistance ratio *SCRR*, and the side channel to the central channel flow rate ratio *SCF* are defined as *S_slp_* = *S_tl_*/(*W_p_* + *W_ps_*), *SCRR* = *R_s_*/ *R_c_*, and *SCF* = *Q_s_*/*Q_c_*, respectively. The parameters *S_tl_* is the change in distance of the side wall from the pillar array plane for a length change of a pillar array period (*W_p_* + *W_ps_*) along the pillar array direction as shown in Figure 1. *R_c_* is the combined hydraulic resistance of the central channels C and C’, and *R_s_* is the hydraulic resistance of the all the side channels S1, S2, S1’, and S2’. However, as the side channels S1 and S2 did not contribute significantly to the net hydraulic resistance *R_s_* due to their larger width, the effective side channel resistance was computed based on the side channels S1’ and S2’ in this paper. Similarly, the net hydraulic resistance of the central channel *R_c_* was effectively due to C’ because of its small width and depth compared to C. *SCF* is computed based on the total flow rate (*Q_s_*) of the side channels S1’ and S2’, and the flow rate (*Q_c_*) through the central channel C’.

Figure 5 shows that there is no significant change in *SCF* with respect to changes in Section II parameters *D_3_*, *W_ps_*, *N*, *S_slp_* and *Q* whereas Figure 6a shows a clear variation of *SCF* with respect to the Section III parameter *SCRR*. This concludes that Section III alone had influence on the net fluid flow through the side channels S1′ and S2′. The reason for this can be understood from the perspective that the fluid flow rate through a channel depends to a great deal on the hydraulic resistance offered by the channel instead of the details of the various geometrical obstructions in the path. The latter observation was further demonstrated experimentally by measuring *SCF* with respect to variations in *SCRR* and has been shown in Figure 6b. The device used for the experiments was chosen to have all the parameters similar to that of the simulated device, except that the number of pillars were taken to be 1000 for the reasons discussed in the next section.

#### 3.1.2. Effect of Section II Parameters [Number of Pillars (*N*) and Side Channel Slope (*S_slp_*)] on the Uniformity of the Flow Across the Pillar Gaps

**Figure 7:**
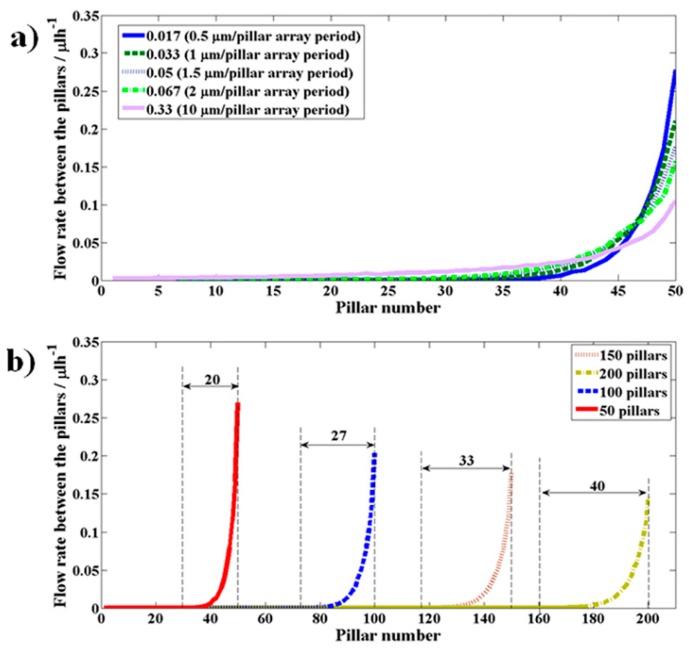
The variation of the flow rate across the pillar gaps, plotted as a function of the pillar number, when the slope of the side channel *S_slp_* (**a**) and number of pillars (*N)* (**b**) are varied.

The simulations were performed on the model described in the simulations section with *D_1_* = 20 μm, *D_2_* = 80 μm, *D_3_* = 5 μm, *SCRR* = 50, and pillar spacing cross-Section 10 μm (width) × 5 μm (depth) while varying the number of pillars (*N*) or the slope of the side channel (*S_slp_*). *SCRR* was kept fixed at a specific value to keep the *SCF* constant throughout the simulations. The choice of *SCRR* to 50 during the simulations has no practical relevance and can be kept at any number to understand the flow behavior. By keeping the number of pillars fixed to 50, the slope of the side channel wall (*S_slp_*) was varied from 0.015 to 0.065 by varying the *S_tl_* from 0.5 μm to 10 μm per pillar array period. The resulting flow rate variation between the pillars as a function of pillar position in the model is shown in Figure 7a.

One observation is that the flow rate distribution across each pillar gap was changing as a function of the position of the pillar. More specifically, this variation is more pronounced towards the end pillars. The flow rate rapidly shoots up towards the end pillars. This is an undesirable feature if this section of the design is expected to extract fluid volumes smaller than the volume of the critical particle across all pillar gaps. As *S_slp_* is increased, a similar variation of the flow rate continued except for one major change. That is, the peak of this flow rate has fallen by redistributing this growing flow rate over larger number of pillars. This implies that the larger the slope of this wall, the more uniform the distribution of the flow rate across the pillars.

Figure 7b shows the variation of the flow rate across the pillar gaps as a function of the number of pillars, for *S_tl_* as 0.5 μm per pillar array period. As the number of pillars has been increased from 50 to 200, the decrease in the peak flow rate and the redistribution of this growing flow rate across the pillars can be clearly seen. The number of pillars over which the variable flow rate is present is determined by finding the pillar at which the flow rate drops to 1% of the peak flow rate and counting the pillars backwards from the pillar at which peak flow rate was observed. These observations suggest that the choice of large values for *S_slp_* and *N* seem to contribute to the increased uniformity of the flow rate across the pillar gaps.

### 3.2. Experimental

Having found relevant parameters that affect the performance of the device from the simulations, the devices were fabricated with those optimum parameters. As the purity of the particle-free fluid was not obtained from our simulations, the experiments were conducted to further probe the performance of the device (purity of particle-free fluid) with respect to other relevant parameters, such as particle size, time, the flow rate, hematocrit and *SCRR*.

#### Variation of Purity of Plasma Generated as a Function of Time, Flow Rate (*Q*), Hematocrit, and *SCRR*

**Figure 8:**
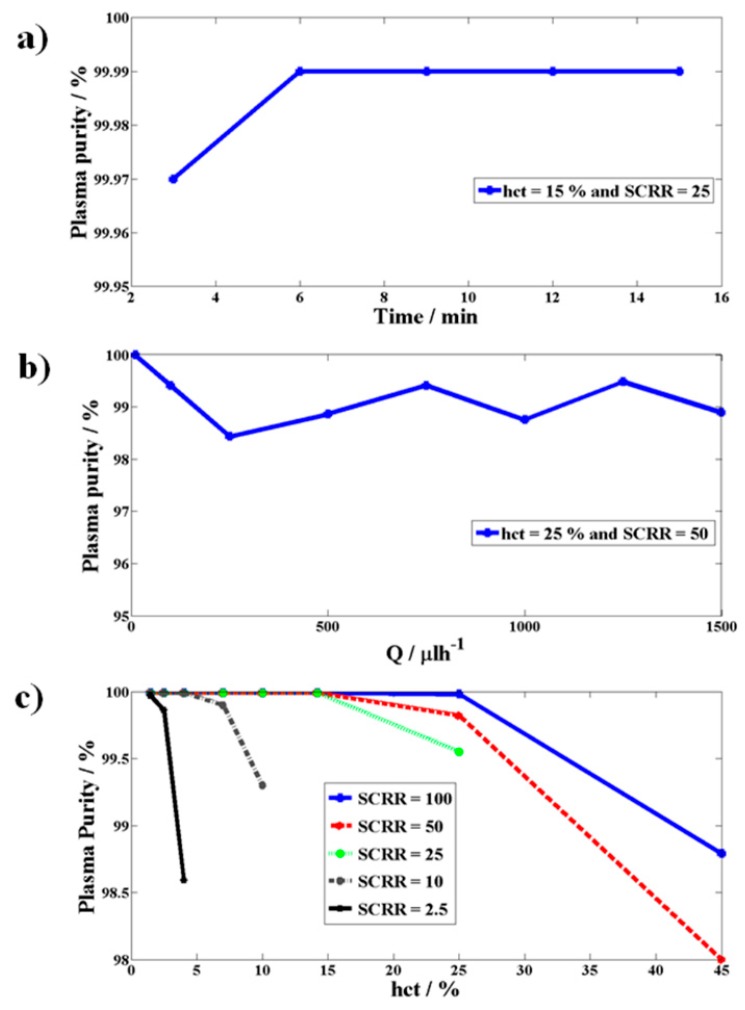
The variation of plasma purity as a function of (**a**) duration of the experiment (time), (**b**) flow rate (*Q*), (**c**) hematocrit (*hct*) and *SCRR*.

The proposed microfluidic device was fabricated with parameters *D_1_* = 20 μm, *D_2_* = 74 μm, *D_3_* = 4.5 μm, *W_ps_* = 12 μm, *W_p_* = 18 μm, *S_tl_* = 0.5 μm and with *SCRR* = 2.5, 10, 25, 50, and 100. The experiments were performed on the fabricated device with respect to time, the flow rate, hematocrit, and *SCRR*, and the purity of plasma collected was evaluated using image processing as discussed in the section on the experimental procedure. Figure 8a shows the time independence of the purity of plasma generated and to a value of close to 100% at a hematocrit of 15% and *SCRR* = 25. This infers that the quality of plasma generated is very pure and is independent of time and can be used anytime during chip analysis.

As can be observed from Figure 8b, plasma purity remains constant at approximately 99% with a maximum variation of only up to 1% with respect to the variation in the flow rate to approximately three orders of magnitude ranging from 10 μlh^−1^ to 1500 μlh^−1^ at a hematocrit of 25% and *SCRR* = 50. This clearly demonstrates the flow rate independent performance of the proposed technique and can be used for a wide range of applications.

**Figure 9:**
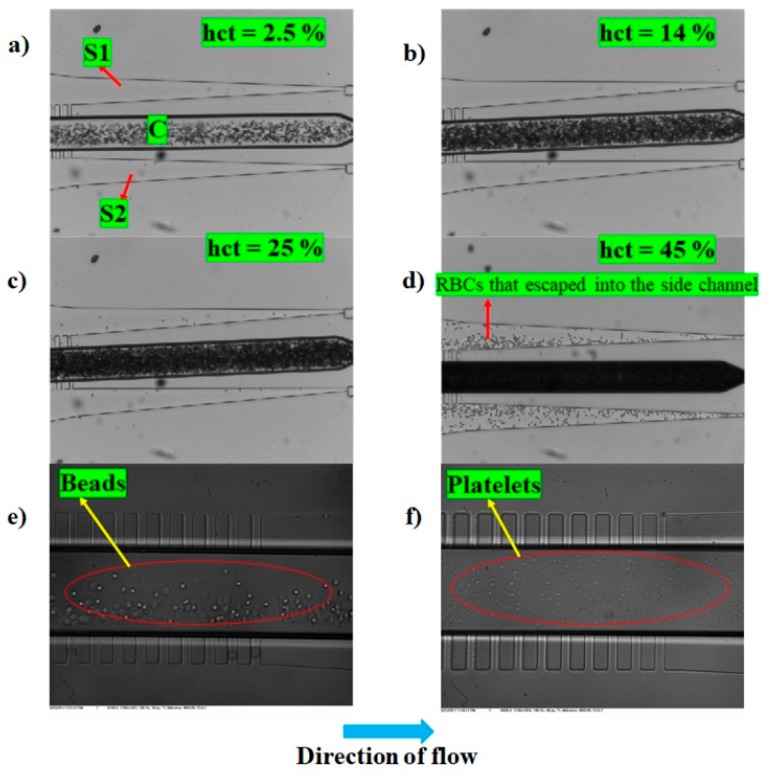
Experimental images illustrating the functioning of the proposed device for various particle concentrations and sizes. (**a**–**d**) represent the experimental images depicting the concentration of RBCs in central channel C and those that escaped into the side channels S1 and S2 for different values of hematocrit. (**a**) 2.5% hematocrit, (**b**) 14% hematocrit, (**c**) 25% hematocrit, and (**d**) 45% hematocrit. (**e**,**f**) represent the experimental images depicting the localization of particles (5 μm beads and platelets) to the central region C alone. The *SCRR* of the devices used in (**a**–**d**) is 100 and in (**e**,**f**) is 50.

Figure 8c shows the variation of plasma purity as a function of hematocrit and *SCRR*. A smaller *SCRR* is expected to provide a larger side channel flow rate as it offers low resistance to the flow inside the channels, however, it may compromise purity at higher hematocrits. Larger *SCRR* is expected to provide a smaller side channel flow rate while offering very high purity even at higher hematocrits. Figure 8c shows experimental results that agree with the theory. The smaller *SCRR* of 2.5 offered 100% purity until 2.5% hematocrit and started dropping until 98.6% as the hematocrit reached 5%. However, larger *SCRR* of 100 offered 100% purity until 28% hematocrit and the purity dropped by only 1% even at a hematocrit of 45%. Figure 9a–d show the images of RBCs at various hematocrit values inside the central channel C and those that escaped into the side channels S1 and S2 at *SCRR* of 100.

The yield of the device (*Y*) is the percentage fraction of the plasma that could be extracted into the side channels from the available plasma in the injected sample and is defined as,

$$Y\;=Q_{s}/\;\left[Q\;\times \;\left(1\;-\;hct\right)]\;=\;SCF/[\left(1+SCF\right)\times \left(1-hct\right)\right],$$

as the true yield also depends on the quantity of the particle-free fluid available. The smaller *SCRR* of 2.5 (at *hct* = 2.5%) has contributed to a plasma yield of approximately 14% and the larger *SCRR* of 100 (at *hct* = 45%) contributed to a smaller plasma yield of approximately 4%. This lower yield at higher hematocrits is not an upper limit to this proposed device, and it can be further increased without compromising on purity, by multiplexing this design in stages and suitably adjusting the side channel resistances as discussed in the subsequent section. Further, 5 μm beads and platelets were pumped at 100 μlh^−1^ into a microfluidic device with *SCRR* = 50, and the purity of the recovered particle-free fluid was close to 100%. Figure 9e,f show the representative images of the beads and platelets inside the microfluidic device close to the end of Section II, respectively. It can be clearly seen that no platelets and beads were observed in the side channels S1 and S2 and the whole of the particles were concentrated in the central channel.

### 3.3. Discussion on the Choice of Parameters for Wee-Extraction

Consider the case in which one value of *SCRR* gave a specific *SCF*. Hence, for a sample flow rate of *Q*, the side channel total flow rate is *Q*_s_ = (*Q* × *SCF)*/(1 + *SCF*). As the device has two side channels S1 and S2, the flow rate through each is half of *Q_s_*. For N number of pillars on each side, the flow rate averaged through each pillar gap is *Q*_pg_ = *Q*_s_/(2 × *N*). For a particle of critical volume *V_p_* not to escape through the pillar gap, the volume being drawn through the pillar gaps should be smaller than *V_p_*. As the particle passes closely adjacent to the pillars, it has two components of velocity *u* and *v*. *u* is along the direction of main sample flow and is responsible for the particles to retain in the central channel, whereas *v* is perpendicular to the direction of main sample flow and into the pillar gaps and is responsible for the particle escape into the side channels S1 and S2. Due to the motion of the particle along main sample flow, each particle spends time duration of approximately *t_pg_* = *W_ps_*/*u*. Within this duration, the volume of fluid that escapes into the pillar gaps is *W_ps_* × *D_3_* × *v* × *t_pg_* = *Q_pg_* × *t_pg_*. When this volume is smaller than *V_p_*, the particles do not escape into the side channels, thereby holding a possibility to achieve 100% purity of the side channel fluid collected. The reduction in the expected purity can arise when this criterion that has been derived for the uniform flow across the pillars is not met.

From the devices used for the experiments, consideration is given to a device with *SCRR* = 100 for analysis. For this *SCRR*, the *SCF* is 2%. For *Q* = 400 μlh^−1^, *W_ps_* = 12 μm, and *N* = 1000, *Q_pg_* will be 0.0039 μlh^−1^. The simulations have been done to estimate *u* at a distance close enough to the pillars (2.5 μm away the center of the pillar and 2.5 μm away from the top of pillar) and was found to be 0.24 mms^−1^. This gave rise to time duration of 50 ms, and the volume of suction as 54 fL per pillar gap. In the case of plasma extraction using whole blood, the quantity of fluid being extracted is smaller than the RBC particle size (100–120 fL), hence avoiding possibility of RBC escape through the pillar gaps.

However, certain considerations must be made in view of the observations from the simulations as shown in Figure 7. The flow will not be uniform across the pillars as was presumed in the above analysis. The flow rate across the pillars close to the end of the array in the direction of the flow can be high compared to the pillars in the beginning of the array. This may lead to the particles not meeting the desired criteria as the fluid volume escaping through the pillar gaps are smaller than the particle volume to be separated towards the end pillars thereby leading to a drop in purity of the fluid collected in the side channels. The fabricated device has 1000 pillars which is five times larger than the number of pillars used in the simulation (200), hence, it is expected to have a better uniformity of the flow rate across the pillar gaps than the simulated results show in Figure 7b. At higher hematocrits (45%) of operation of the device, the purity has slightly come down to 99%. This may be an indicator that the flow rate across the pillar gaps towards the end of the array is still larger than the volume of RBC. Increasing the number of pillars and the slope *S_slp_* should help bring down this peak volume that is being extracted across the pillar gaps to smaller than the volume of RBC, thereby providing 100% purity even at much larger hematocrits (> 45%).

From Figure 5e, it can be observed that *SCF* remains unchanged as the sample flow rate is increased, indicating that the flow rate in the side channels and central channel proportionately increase. On an average, this indicates how proportionately *u* and *v* change, thereby leading to almost similar flow volumes across the pillar gaps that are independent of the sample flow rate. This also suggests how the purity can remain almost constant even for a wide variation of flow rates. This behavior can be observed experimentally as shown in Figure 8b. The above analysis is very general and can be applied to separating particles of any size, volume and concentration at any desired flow rate. Suitably choosing the large values for *SCRR*, number of pillars *N* (>1000), and the slope of the side channels *S_slp_* (*S_tl_* > 0.5 μm per period of the array), and smaller height *D_3_* and spacing between the pillars *W_ps_* as per the above guidelines. This assists in designing a device that can separate particles smaller than platelets (<2 μm), without the need for complicated fabrication, and at dimensions that can be easily fabricated using the conventional techniques of microfabrication. The yield of this proposed device can be increased by multiple folds and a way of accomplishing this is presented in the following section.

### 3.4. Enhancing the Yield through Multiplexing

**Figure 10:**
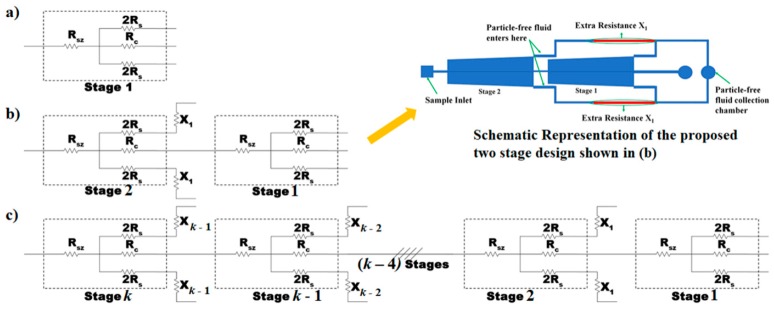
Schematic illustrating the strategies to enhance the yield. (**a**) Hydraulic resistance model of the device along with the side and central channels C’, S1’ and S2’ and is depicted as Stage-I. (**b**) Hydraulic resistance model when Stage-II being added to Stage-I and the representative schematic is shown in the inset. (**c**) Hydraulic resistance model when *k* such stages are present in the device.

Consider one of the proposed designs that have been discussed so far as stage 1 and for example, it produces a yield of *Y* as shown in the resistance model of the device, Figure 10a. If this stage (stage 1) is added to the end of a similar such stage (stage 2), it adds additional resistance to the central channel of stage 2, thereby reducing the purity of the plasma collected. To ensure that the *SCRR* of the stage 2 of the newly formed combination device remains at its earlier value, an additional resistance *X_1_* needs to be added on either side of stage 2, as shown in Figure 10b. This process ensures the increment in the yield by a factor of [2 − *SCF*/(1 + *SCF*)], as the two stages contribute to the plasma generation. The inset in Figure 10 shows a representative schematic of the device with stages 1 and 2 as presented in Figure 10b. Similarly, for a *k*-stage device, the yield is expected to be:

$$\left[k+\frac{-SCF}{1+SCF}+{\left(\frac{-SCF}{1+SCF}\right)}^{2}+{\left(\frac{-SCF}{1+SCF}\right)}^{3}+\;\ldots \;+\;{\left(\frac{-SCF}{1+SCF}\right)}^{\left(k-1\right)}\right]\times Y,$$

when ensured that the purity is unchanged by adding extra resistances to each stage of the device (Figure 10c). As an example, the yield of the device with *SCRR* of 100 at hematocrit of 45% is 4% and this can be increased to approximately 20% by suitably cascading five such stages, considering the extra resistances that need to be incorporated during the design. In this analysis, it was presumed that the yield of each stage cannot change substantially with respect to the addition of a greater number of stages. However, it is noted that in cases when the yield of each stage changes substantially, which typically may happen at lower *hct* and higher *SCF*, the modified yield of each stage needs to be considered to arrive at an accurate value of the total yield.

## 4. Conclusions

This paper presents a technique that can, in principle, separate the particles and the fluid in 100% purity and higher yields for a wide range of particle sizes, concentrations, and flow rates. The principle of operation was studied through the theory and simulations, while the proof-of-principle was demonstrated using 5 μm beads, platelets, and blood of ranging hematocrits (2.5%–45%). Lower hematocrit (2.5%) blood was separated in 100% purity and high yields (14%), whereas the high hematocrit blood (45%) was separated in purities ranging from 98% to 100% depending upon the flow rate of operation (10 μlh^−1^–1500 μlh^−1^). The yield can be increased in approximately *k*-folds without compromising purity by cascading the proposed design *k*-times appropriately. The purity can be enhanced further for any size and the concentration of particles by suitably choosing optimum values for the side channel to the central channel hydraulic resistance ratio (*SCRR*), the number of pillars (*N*), the slope (*S_slp_*), the pillar spacing (*W_ps_*), and the height of the pillars (*D_3_*).

Unique features of this technique are the stepped pillar arrays and the hydraulic resistance tuners. Stepped pillar arrays play a unique and important role in extracting smaller quantities of fluid through the pillar gaps by providing the smaller cross-section for the fluid flow into the side channels. Another associated important feature of these stepped pillar arrays is that the pillar spacing cross-section can be much larger than the size of the particle to be separated from the particle-free fluid, thereby facilitating ease in fabrication even for separating submicron particles. This was very well demonstrated by choosing the pillar gap cross-section as 12 μm × 4.5 μm and the particles to be separated as platelets (2 μm–4 μm) with 100% purity. The hydraulic resistance tuners majorly control the net yield and play a major role in deciding the critical particle volume to be decantated in association with the stepped pillar arrays. The stepped pillar arrays along with hydraulic resistance tuners facilitate the flow-rate-independent the fluid flow through the pillar gaps, thereby ensuring the flow-rate-independent application of this technique. This was demonstrated for flow rates ranging from 10 μlh^−1^–1500 μlh^−1^.

This novel technique offers several potential applications towards plasma separation from whole blood for biochemical tests to achieve self-sheath flow by utilizing the particle-free fluid to surround the central particle-rich sample, and the enrichment of rare cells such as circulating tumor cells (CTCs) for cancer detection. As a summary, this paper offers the design, theory, and guidelines to achieve the decantation of the particles of any size and concentration at 100% purity and desired yield, while the in-flow at any flow rate of operation concurrently provides great ease of fabrication.

## Supplementary Materials

The following are available online at https://www.mdpi.com/2072-666X/10/7/471/s1, Figure S1: Mask design for layer-1 that was used to fabricate the Master. Figure S2: Mask design for layer-2 that was used to fabricate the Master. Figure S3: Mask design for layer-3 that was used to fabricate the Master. Figure S4: Schematic of the device design that was used for performing simulations.
