# Supplementary material for: Microfluidic In-Flow Decantation Technique Using Stepped Pillar Arrays and Hydraulic Resistance Tuners
Source: Micromachines (Basel). 2019 Jul 15;10(7):471. doi: 10.3390/mi10070471 (PMC6680991; doi:10.3390/mi10070471)
Supplement: Supplementary file 1 [file micromachines-10-00471-s001.pdf]

Supplementary Materials: Microfluidic in-flow Decantation Technique using Stepped Pillar Arrays and Hydraulic Resistance Tuners

Gangadhar Eluru, Pavan Nagendra and Sai Siva Gorthi

Mask designs:

Screenshots of the mask designs that were used to fabricate the device are shown below.

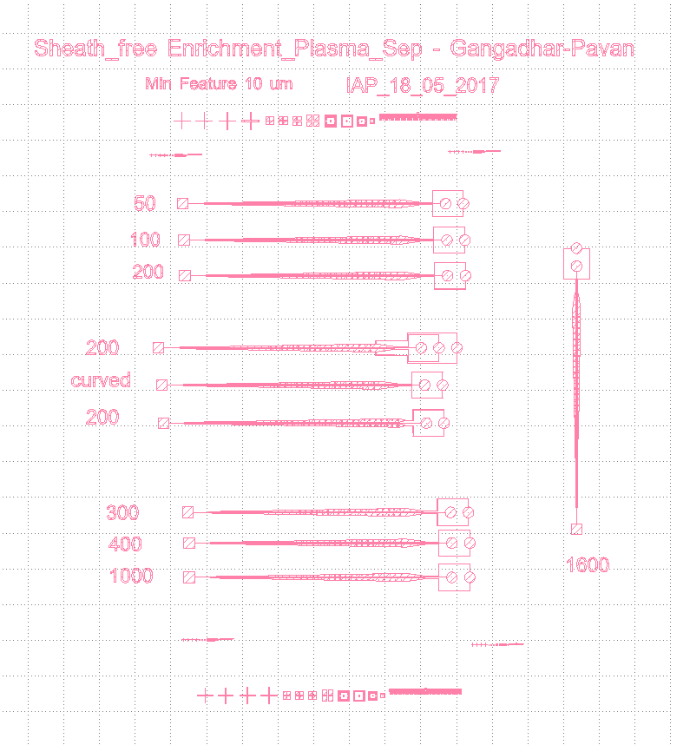

Figure S1. Mask design for layer-1 that was used to fabricate the Master.

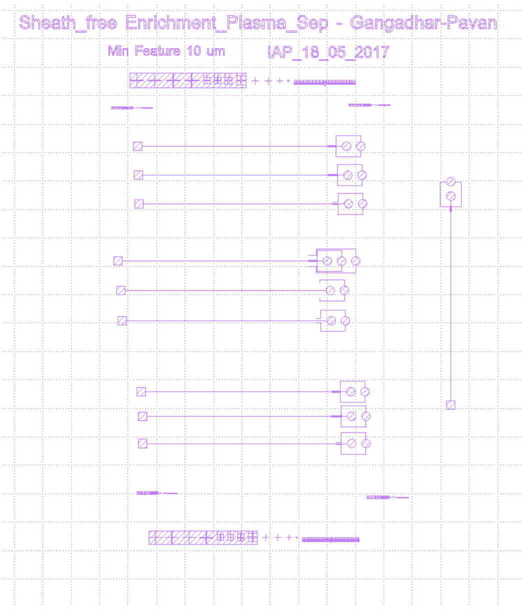

Figure S2. Mask design for layer-2 that was used to fabricate the Master.

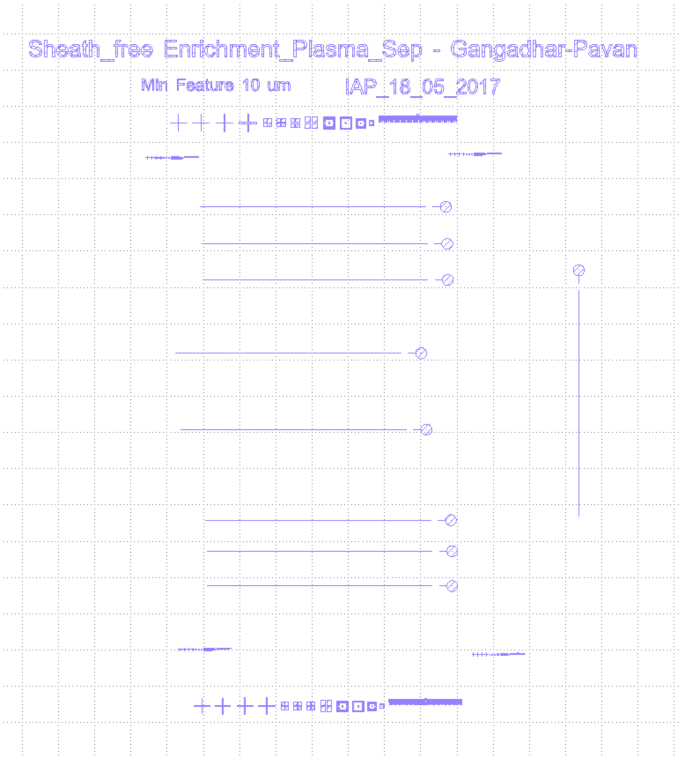

Figure S3. Mask design for layer-3 that was used to fabricate the Master.

Simulated Design:

Schematic representing the design that was used for simulations is shown below.

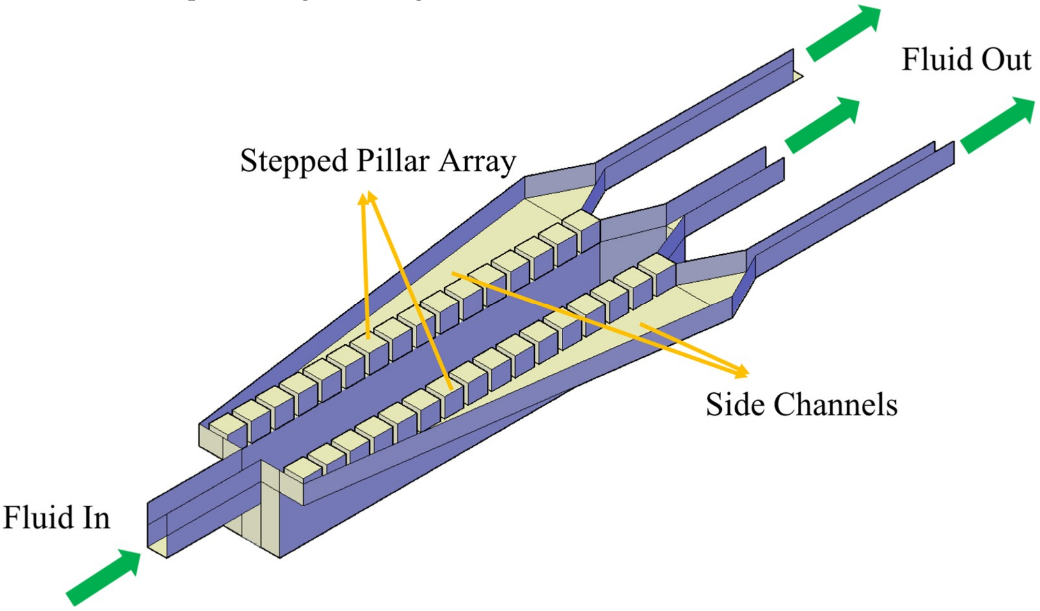

Figure S4. Schematic of the device design that was used for performing simulations.

MATLAB Code for Data Analysis:

MATLAB code that was used for detecting and counting the red blood cells in the side channels is given below.

```
clc ;
close all;
```

```
clear all;
%%
vidData=VideoReader('filename.avi');
noFrame=vidData.NumberOfFrames;
% noFrame=15000;
mask1=roipoly((read(vidData, 1)));
%%
mask2=roipoly((read(vidData, 2)));

%% Generate Background
bg = 0;
count = 10 ;% set number of frame to be averaged
N = 1; % start frame number
te=0;

for i = N:N+count
    temp=((read(vidData, i)));
    te = temp.*uint8(mask1);
    te=cat(1,te,temp.*uint8(mask2));
    bg=bg+(te);
    te=0;
end

bg   = (bg /(count+1));

%% subtract all subsequent frames and write into a video.
sum=0;
for k =1:noFrame

  Copy=(read(vidData, k));
  CurrFrame = double(read(vidData, k));

    CurrFrame1 = Copy.*uint8(mask1);
      CurrFrame1=cat(1,CurrFrame1,Copy.*uint8(mask2));

    CurrFrame2 = CurrFrame.*double(mask1);
      CurrFrame2=cat(1,CurrFrame2,CurrFrame.*double(mask2));

  Sub = double(CurrFrame2(:,:)-double(bg(:,:)));
  Submin = min(Sub(:));
  Submax = max(Sub(:));
  AdjBGSub = uint8( (Sub - Submin)/(Submax-Submin) * 255);
```

```
I = AdjBGSub;
  [A, B]=size(I);

[~, threshold] = edge(I, 'sobel');
fudgeFactor = 1.3;
BWs = edge(I,'sobel', threshold * fudgeFactor);
se90 = strel('line', 2, 90);
se0 = strel('line', 2, 0);
BWsdil = imdilate(BWs, [se90 se0]);
BWdfill = imfill(BWsdil, 'holes');
BWdfillopen = imopen(BWdfill,strel('disk',2,4));
BWnobord = imclearborder(BWdfillopen, 4);
seD = strel('disk',2,4);
BWfinal = imerode(BWnobord,seD);

CC =bwconncomp(BWfinal);
Centroids=regionprops(CC,'Centroid');
sum=sum+CC.NumObjects;
end
```
